# CsMgPO_4_

**DOI:** 10.1107/S1600536809025434

**Published:** 2009-07-04

**Authors:** Nataliya Yu. Strutynska, Igor V. Zatovsky, Vyacheslav N. Baumer, Nikolay S. Slobodyanik

**Affiliations:** a Department of Inorganic Chemistry, Taras Shevchenko National University, 64 Volodymyrska Street, 01601 Kyiv, Ukraine; b STC ‘Institute for Single Crystals’, NAS of Ukraine, 60 Lenin Avenue, 61001 Kharkiv, Ukraine

## Abstract

Caesium magnesium orthophosphate is built up from MgO_4_ and PO_4_ tetra­hedra (both with . *m*. symmetry) linked together by corners, forming a three-dimensional framework. The Cs atoms have .*m*. site symmetry and are located in hexa­gonal channels running along the *a*- and *b*-axis directions.

## Related literature

For the properties of double phosphates *A* ^I^ *B* ^II^PO_4_ (*A* ^I^ = alkali metal; *B* ^II^ = Ca, Sr, Ba, Zn, Cd, Pb) such as ferroelectric and non-linear optical behaviour, see: Blum *et al.* (1984 ▶); Elouadi *et al*. (1984 ▶); Sawada *et al*. (2003 ▶). Several polymorphs have been found among orthophosphates containing Cs and divalent metals, see: Blum *et al.* (1986 ▶) for CsZnPO_4_. In contrast, CsMnPO_4_ occurs in only one type, see: Yakubovich *et al.* (1990 ▶). The title compound is isotypic with the *Pnma* form of CsZnPO_4_. For related structures, see: Yakubovich *et al.* (1990 ▶); Blum *et al.* (1986 ▶); Zaripov *et al.* (2008 ▶).

## Experimental

### 

#### Crystal data

CsMgPO_4_
*M* *_r_* = 252.19
Orthorhombic, 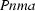

*a* = 8.9327 (2) Å
*b* = 5.5277 (2) Å
*c* = 9.6487 (3) Å
*V* = 476.43 (3) Å^3^
*Z* = 4
Mo *K*α radiation
μ = 8.13 mm^−1^
*T* = 293 K
0.12 × 0.10 × 0.08 mm

#### Data collection

Oxford Diffraction Xcalibur-3 diffractometer
Absorption correction: multi-scan (Blessing, 1995 ▶) *T*_min_ = 0.413, *T*_max_ = 0.503
8753 measured reflections
1137 independent reflections
874 reflections with *I* > 2σ(*I*)
*R*_int_ = 0.027

#### Refinement

*R*[*F* ^2^ > 2σ(*F* ^2^)] = 0.021
*wR*(*F* ^2^) = 0.047
*S* = 1.00
1137 reflections
41 parameters
Δρ_max_ = 1.23 e Å^−3^
Δρ_min_ = −1.02 e Å^−3^

### 

Data collection: *CrysAlis CCD* (Oxford Diffraction, 2006 ▶); cell refinement: *CrysAlis CCD*; data reduction: *CrysAlis RED* (Oxford Diffraction, 2006 ▶); program(s) used to solve structure: *SHELXS97* (Sheldrick, 2008 ▶); program(s) used to refine structure: *SHELXL97* (Sheldrick, 2008 ▶); molecular graphics: *DIAMOND* (Brandenburg, 1999 ▶); software used to prepare material for publication: *WinGX* (Farrugia, 1999 ▶) and *enCIFer* (Allen *et al.*, 2004 ▶).

## Supplementary Material

Crystal structure: contains datablocks global, I. DOI: 10.1107/S1600536809025434/mg2075sup1.cif

Structure factors: contains datablocks I. DOI: 10.1107/S1600536809025434/mg2075Isup2.hkl

Additional supplementary materials:  crystallographic information; 3D view; checkCIF report

## Figures and Tables

**Table 1:** Selected bond lengths (Å)

Cs1—O2	3.1951 (9)
Cs1—O2^i^	3.2166 (9)
Cs1—O3^ii^	3.4476 (11)
Cs1—O1^i^	3.5224 (11)
Cs1—O1^ii^	3.6496 (11)
Cs1—O3^iii^	3.6968 (18)
Mg1—O1	1.8847 (13)
Mg1—O3^iv^	1.8932 (13)
Mg1—O2^v^	1.9228 (8)
P1—O1	1.5056 (13)
P1—O3	1.5138 (13)
P1—O2	1.5249 (8)

Symmetry codes: (i) 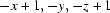; (ii) 
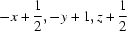
; (iii) 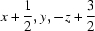; (iv) 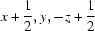; (v) 
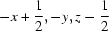
.

**Fig. 1.**
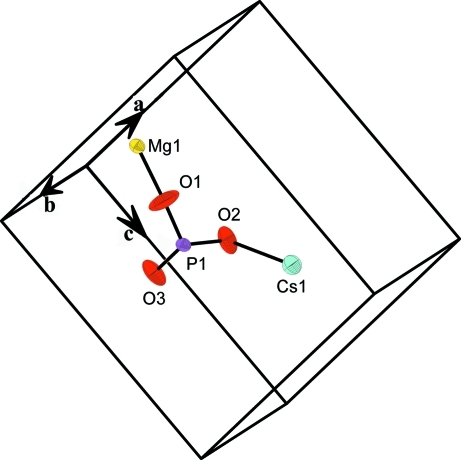
View of CsMgPO4 with displacement ellipsoids at the 50% probability level.

**Fig. 2.**
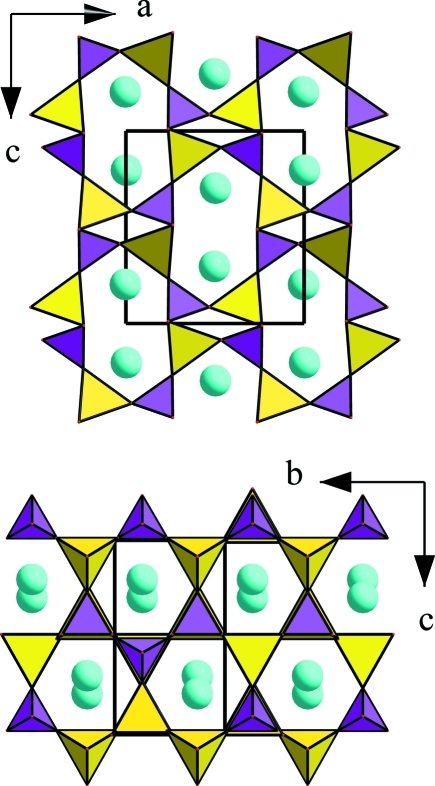
Formation of hexagonal channels along a and b directions in CsMgPO4 (PO4, pink; MgO4, yellow; Cs, blue).

## supplementary crystallographic information

### Comment

Double phosphates A^I^B^II^PO_4_ (A^I^ = alkali metal; B^II^ = Ca, Sr, Ba, Zn, Cd, Pb) exhibit important properties such as ferroelectric and nonlinear optical behaviour (Blum *et al.*, 1984; Elouadi *et al.*, 1984; Sawada *et al.*, 2003). Among some orthophosphates containing Cs and divalent metals, several polymorphs have been found. For instance, CsZnPO_4_ occurs in a monoclinic (space group *P*2_1_/*a*) and two orthorhombic types (space groups *Pna*2_1_ and *Pnma*) (Blum *et al.*, 1986). In contrast, CsMnPO_4_ occurs in only one type (space group *Pna*2_1_) (Yakubovich *et al.*, 1990). CsMgPO_4_, reported here, is isotypic with the *Pnma* form of CsZnPO_4_.

Except for O2 (8d), all atoms are in special positions (4c) (Fig. 1). Each MgO_4_ tetrahedron is linked with four PO_4_ tetrahedra *via* common vertices, resulting in a three-dimensional framework with two types of hexagonal channels, filled by Cs atoms, along the a and b directions (Fig. 2). With a cut-off distance of 3.7 Å, the Cs atoms are 11-coordinate. In general, the principles of crystal structure building are equivalent to those in Cs*M*^II^PO_4_ (*M*^II^ = Mn, Zn) (Yakubovich *et al.*, 1990; Blum *et al.*, 1986) and CsLi_0.5_Al_0.5_PO_4_ (Zapirov *et al.*, 2008).

### Experimental

In the course of investigating the Cs_2_O–MgO–Bi_2_O_3_–P_2_O_5_ system, the starting components CsPO_3_ (3.0 g), MgO (0.113 g) and Bi_2_O_3_ (0.652 g) were finely ground and melted in a platinum crucible at 1273 K. The melt was kept at this temperature over 2 h to reach homogeneity and then cooled at a rate of 30 K h^-1^ to 993 K. After the melt was cooled to room temperature and treated with a small amount of deionized water, colorless needle-shaped crystals were isolated. X-ray powder diffraction showed that CsMgPO_4_ is the only crystalline product.

### Refinement

The deepest hole and the highest peak are 0.67 Å and 0.65 Å, respectively, from Cs1.

### Crystal data

**Table:**

CsMgPO_4_	*F*(000) = 456
*M**_r_* = 252.19	*D*_x_ = 3.516 Mg m^−^^3^
Orthorhombic, *P**n**m**a*	Mo *K*α radiation, λ = 0.71073 Å
Hall symbol: -P 2ac 2n	Cell parameters from 8753 reflections
*a* = 8.9327 (2) Å	θ = 3.1–35.0°
*b* = 5.5277 (2) Å	µ = 8.13 mm^−^^1^
*c* = 9.6487 (3) Å	*T* = 293 K
*V* = 476.43 (3) Å^3^	Prism, colorless
*Z* = 4	0.12 × 0.10 × 0.08 mm

### Data collection

**Table:**

Oxford Diffraction Xcalibur-3 diffractometer	1137 independent reflections
Radiation source: fine-focus sealed tube	874 reflections with *I* > 2σ(*I*)
graphite	*R*_int_ = 0.027
φ and ω scans	θ_max_ = 35.0°, θ_min_ = 3.1°
Absorption correction: multi-scan (Blessing, 1995)	*h* = −14→14
*T*_min_ = 0.413, *T*_max_ = 0.503	*k* = −8→8
8753 measured reflections	*l* = −15→15

### Refinement

**Table:**

Refinement on *F*^2^	Primary atom site location: structure-invariant direct methods
Least-squares matrix: full	Secondary atom site location: difference Fourier map
*R*[*F*^2^ > 2σ(*F*^2^)] = 0.021	*w* = 1/[σ^2^(*F*_o_^2^) + (0.0265*P*)^2^] where *P* = (*F*_o_^2^ + 2*F*_c_^2^)/3
*wR*(*F*^2^) = 0.047	(Δ/σ)_max_ = 0.018
*S* = 1.00	Δρ_max_ = 1.23 e Å^−^^3^
1137 reflections	Δρ_min_ = −1.02 e Å^−^^3^
41 parameters	Extinction correction: *SHELXL97* (Sheldrick, 2008)
0 restraints	Extinction coefficient: 0.0211 (4)

### Special details

**Table:**

Geometry. All e.s.d.'s (except the e.s.d. in the dihedral angle between two l.s. planes) are estimated using the full covariance matrix. The cell e.s.d.'s are taken into account individually in the estimation of e.s.d.'s in distances, angles and torsion angles; correlations between e.s.d.'s in cell parameters are only used when they are defined by crystal symmetry. An approximate (isotropic) treatment of cell e.s.d.'s is used for estimating e.s.d.'s involving l.s. planes.
Refinement. Refinement of *F*^2^ against ALL reflections. The weighted *R*-factor *wR* and goodness of fit *S* are based on *F*^2^, conventional *R*-factors *R* are based on *F*, with *F* set to zero for negative *F*^2^. The threshold expression of *F*^2^ > σ(*F*^2^) is used only for calculating *R*-factors(gt) *etc*. and is not relevant to the choice of reflections for refinement. *R*-factors based on *F*^2^ are statistically about twice as large as those based on *F*, and *R*- factors based on ALL data will be even larger.

### Fractional atomic coordinates and isotropic or equivalent isotropic displacement parameters (Å^2^)

**Table:**

	*x*	*y*	*z*	*U*_iso_*/*U*_eq_	
Cs1	0.497176 (11)	0.25	0.703332 (10)	0.02472 (2)	
Mg1	0.32166 (5)	0.25	0.08109 (5)	0.01434 (11)	
P1	0.20302 (4)	0.25	0.41474 (4)	0.01345 (7)	
O1	0.26034 (19)	0.25	0.26799 (13)	0.0590 (6)	
O2	0.26291 (11)	0.02604 (13)	0.48850 (9)	0.0328 (2)	
O3	0.03356 (14)	0.25	0.41501 (19)	0.0345 (4)	

### Atomic displacement parameters (Å^2^)

**Table:**

	*U*^11^	*U*^22^	*U*^33^	*U*^12^	*U*^13^	*U*^23^
Cs1	0.02161 (4)	0.02632 (5)	0.02623 (4)	0	−0.00031 (4)	0
Mg1	0.01301 (19)	0.0133 (2)	0.0168 (2)	0	0.00166 (17)	0
P1	0.01272 (13)	0.01259 (14)	0.01505 (14)	0	0.00207 (12)	0
O1	0.0594 (11)	0.1014 (17)	0.0162 (7)	0	0.0121 (7)	0
O2	0.0239 (4)	0.0190 (4)	0.0555 (6)	−0.0002 (3)	−0.0016 (4)	0.0156 (4)
O3	0.0121 (4)	0.0286 (6)	0.0629 (10)	0	−0.0003 (6)	0

### Geometric parameters (Å, °)

**Table:**

Cs1—O2	3.1951 (9)	Mg1—Cs1^xiii^	4.1402 (4)
Cs1—O2^i^	3.1951 (9)	P1—O1	1.5056 (13)
Cs1—O2^ii^	3.2166 (9)	P1—O3	1.5138 (13)
Cs1—O2^iii^	3.2166 (9)	P1—O2^i^	1.5249 (8)
Cs1—O3^iv^	3.4476 (11)	P1—O2	1.5249 (8)
Cs1—O3^v^	3.4476 (11)	P1—Cs1^ix^	3.8727 (3)
Cs1—O1^vi^	3.5224 (11)	P1—Cs1^xiii^	3.8727 (3)
Cs1—O1^ii^	3.5224 (11)	P1—Cs1^vi^	4.0136 (3)
Cs1—O1^v^	3.6496 (11)	P1—Cs1^ii^	4.0136 (3)
Cs1—O1^iv^	3.6496 (11)	P1—Cs1^xiv^	4.1184 (4)
Cs1—O3^vii^	3.6968 (18)	O1—Cs1^vi^	3.5224 (11)
Cs1—Mg1^vi^	3.8189 (4)	O1—Cs1^ii^	3.5224 (11)
Mg1—O1	1.8847 (13)	O1—Cs1^ix^	3.6496 (11)
Mg1—O3^viii^	1.8932 (13)	O1—Cs1^xiii^	3.6496 (11)
Mg1—O2^ix^	1.9228 (8)	O2—Mg1^v^	1.9228 (8)
Mg1—O2^x^	1.9228 (8)	O2—Cs1^ii^	3.2166 (9)
Mg1—Cs1^vi^	3.8189 (4)	O3—Mg1^xii^	1.8932 (13)
Mg1—Cs1^ii^	3.8189 (4)	O3—Cs1^ix^	3.4476 (11)
Mg1—Cs1^xi^	3.9678 (5)	O3—Cs1^xiii^	3.4476 (11)
Mg1—Cs1^xii^	3.9916 (5)	O3—Cs1^xiv^	3.6968 (18)
Mg1—Cs1^ix^	4.1402 (4)		
			
O2—Cs1—O2^i^	45.59 (3)	O1—Mg1—Cs1^ix^	61.80 (3)
O2—Cs1—O2^ii^	83.06 (3)	O3^viii^—Mg1—Cs1^ix^	133.01 (2)
O2^i^—Cs1—O2^ii^	104.291 (19)	O2^ix^—Mg1—Cs1^ix^	48.11 (3)
O2—Cs1—O2^iii^	104.291 (19)	O2^x^—Mg1—Cs1^ix^	113.07 (3)
O2^i^—Cs1—O2^iii^	83.06 (3)	Cs1^vi^—Mg1—Cs1^ix^	128.277 (13)
O2^ii^—Cs1—O2^iii^	56.64 (3)	Cs1^ii^—Mg1—Cs1^ix^	69.709 (5)
O2—Cs1—O3^iv^	130.00 (3)	Cs1^xi^—Mg1—Cs1^ix^	122.243 (9)
O2^i^—Cs1—O3^iv^	91.24 (3)	Cs1^xii^—Mg1—Cs1^ix^	72.323 (7)
O2^ii^—Cs1—O3^iv^	140.73 (3)	O1—Mg1—Cs1^xiii^	61.80 (3)
O2^iii^—Cs1—O3^iv^	90.78 (3)	O3^viii^—Mg1—Cs1^xiii^	133.01 (2)
O2—Cs1—O3^v^	91.24 (3)	O2^ix^—Mg1—Cs1^xiii^	113.07 (3)
O2^i^—Cs1—O3^v^	130.00 (3)	O2^x^—Mg1—Cs1^xiii^	48.11 (3)
O2^ii^—Cs1—O3^v^	90.78 (3)	Cs1^vi^—Mg1—Cs1^xiii^	69.709 (5)
O2^iii^—Cs1—O3^v^	140.73 (3)	Cs1^ii^—Mg1—Cs1^xiii^	128.277 (14)
O3^iv^—Cs1—O3^v^	106.58 (5)	Cs1^xi^—Mg1—Cs1^xiii^	122.243 (9)
O2—Cs1—O1^vi^	139.27 (2)	Cs1^xii^—Mg1—Cs1^xiii^	72.323 (7)
O2^i^—Cs1—O1^vi^	98.61 (3)	Cs1^ix^—Mg1—Cs1^xiii^	83.758 (9)
O2^ii^—Cs1—O1^vi^	90.44 (3)	O1—P1—O3	109.98 (10)
O2^iii^—Cs1—O1^vi^	42.55 (2)	O1—P1—O2^i^	108.64 (5)
O3^iv^—Cs1—O1^vi^	51.20 (3)	O3—P1—O2^i^	110.49 (5)
O3^v^—Cs1—O1^vi^	129.16 (3)	O1—P1—O2	108.64 (5)
O2—Cs1—O1^ii^	98.61 (3)	O3—P1—O2	110.49 (5)
O2^i^—Cs1—O1^ii^	139.27 (2)	O2^i^—P1—O2	108.56 (7)
O2^ii^—Cs1—O1^ii^	42.55 (2)	O1—P1—Cs1	116.78 (7)
O2^iii^—Cs1—O1^ii^	90.44 (3)	O3—P1—Cs1	133.24 (7)
O3^iv^—Cs1—O1^ii^	129.16 (3)	O2^i^—P1—Cs1	54.54 (3)
O3^v^—Cs1—O1^ii^	51.20 (3)	O2—P1—Cs1	54.54 (3)
O1^vi^—Cs1—O1^ii^	103.38 (4)	O1—P1—Cs1^ix^	70.23 (4)
O2—Cs1—O1^v^	53.46 (2)	O3—P1—Cs1^ix^	62.56 (4)
O2^i^—Cs1—O1^v^	89.52 (3)	O2^i^—P1—Cs1^ix^	170.83 (4)
O2^ii^—Cs1—O1^v^	99.15 (2)	O2—P1—Cs1^ix^	80.12 (3)
O2^iii^—Cs1—O1^v^	151.35 (2)	Cs1—P1—Cs1^ix^	134.428 (4)
O3^iv^—Cs1—O1^v^	117.11 (3)	O1—P1—Cs1^xiii^	70.23 (4)
O3^v^—Cs1—O1^v^	40.66 (3)	O3—P1—Cs1^xiii^	62.56 (4)
O1^vi^—Cs1—O1^v^	165.554 (5)	O2^i^—P1—Cs1^xiii^	80.12 (3)
O1^ii^—Cs1—O1^v^	77.288 (3)	O2—P1—Cs1^xiii^	170.83 (4)
O2—Cs1—O1^iv^	89.52 (3)	Cs1—P1—Cs1^xiii^	134.428 (4)
O2^i^—Cs1—O1^iv^	53.46 (2)	Cs1^ix^—P1—Cs1^xiii^	91.069 (8)
O2^ii^—Cs1—O1^iv^	151.35 (2)	O1—P1—Cs1^vi^	60.41 (4)
O2^iii^—Cs1—O1^iv^	99.15 (2)	O3—P1—Cs1^vi^	131.89 (3)
O3^iv^—Cs1—O1^iv^	40.66 (3)	O2^i^—P1—Cs1^vi^	48.65 (4)
O3^v^—Cs1—O1^iv^	117.11 (3)	O2—P1—Cs1^vi^	117.22 (4)
O1^vi^—Cs1—O1^iv^	77.288 (3)	Cs1—P1—Cs1^vi^	75.434 (6)
O1^ii^—Cs1—O1^iv^	165.554 (5)	Cs1^ix^—P1—Cs1^vi^	130.546 (10)
O1^v^—Cs1—O1^iv^	98.45 (4)	Cs1^xiii^—P1—Cs1^vi^	70.558 (4)
O2—Cs1—O3^vii^	134.74 (2)	O1—P1—Cs1^ii^	60.41 (4)
O2^i^—Cs1—O3^vii^	134.74 (2)	O3—P1—Cs1^ii^	131.89 (3)
O2^ii^—Cs1—O3^vii^	120.97 (2)	O2^i^—P1—Cs1^ii^	117.22 (4)
O2^iii^—Cs1—O3^vii^	120.97 (2)	O2—P1—Cs1^ii^	48.65 (4)
O3^iv^—Cs1—O3^vii^	54.33 (3)	Cs1—P1—Cs1^ii^	75.434 (6)
O3^v^—Cs1—O3^vii^	54.33 (3)	Cs1^ix^—P1—Cs1^ii^	70.558 (4)
O1^vi^—Cs1—O3^vii^	82.40 (2)	Cs1^xiii^—P1—Cs1^ii^	130.546 (10)
O1^ii^—Cs1—O3^vii^	82.40 (2)	Cs1^vi^—P1—Cs1^ii^	87.043 (8)
O1^v^—Cs1—O3^vii^	83.40 (2)	O1—P1—Cs1^xiv^	173.36 (7)
O1^iv^—Cs1—O3^vii^	83.40 (2)	O3—P1—Cs1^xiv^	63.38 (7)
O2—Cs1—Mg1^vi^	155.689 (15)	O2^i^—P1—Cs1^xiv^	74.87 (4)
O2^i^—Cs1—Mg1^vi^	110.517 (14)	O2—P1—Cs1^xiv^	74.87 (4)
O2^ii^—Cs1—Mg1^vi^	111.987 (18)	Cs1—P1—Cs1^xiv^	69.857 (6)
O2^iii^—Cs1—Mg1^vi^	71.807 (16)	Cs1^ix^—P1—Cs1^xiv^	105.375 (7)
O3^iv^—Cs1—Mg1^vi^	29.64 (2)	Cs1^xiii^—P1—Cs1^xiv^	105.375 (7)
O3^v^—Cs1—Mg1^vi^	106.93 (3)	Cs1^vi^—P1—Cs1^xiv^	123.498 (7)
O1^vi^—Cs1—Mg1^vi^	29.40 (2)	Cs1^ii^—P1—Cs1^xiv^	123.498 (7)
O1^ii^—Cs1—Mg1^vi^	105.34 (2)	P1—O1—Mg1	177.02 (12)
O1^v^—Cs1—Mg1^vi^	136.22 (2)	P1—O1—Cs1^vi^	97.77 (4)
O1^iv^—Cs1—Mg1^vi^	68.03 (2)	Mg1—O1—Cs1^vi^	84.06 (4)
O3^vii^—Cs1—Mg1^vi^	54.563 (11)	P1—O1—Cs1^ii^	97.77 (4)
O1—Mg1—O3^viii^	105.76 (8)	Mg1—O1—Cs1^ii^	84.06 (4)
O1—Mg1—O2^ix^	109.29 (4)	Cs1^vi^—O1—Cs1^ii^	103.38 (4)
O3^viii^—Mg1—O2^ix^	113.71 (4)	P1—O1—Cs1^ix^	86.93 (5)
O1—Mg1—O2^x^	109.29 (4)	Mg1—O1—Cs1^ix^	91.12 (4)
O3^viii^—Mg1—O2^x^	113.71 (4)	Cs1^vi^—O1—Cs1^ix^	174.41 (4)
O2^ix^—Mg1—O2^x^	105.04 (6)	Cs1^ii^—O1—Cs1^ix^	78.860 (3)
O1—Mg1—Cs1^vi^	66.55 (3)	P1—O1—Cs1^xiii^	86.93 (5)
O3^viii^—Mg1—Cs1^vi^	64.25 (3)	Mg1—O1—Cs1^xiii^	91.12 (4)
O2^ix^—Mg1—Cs1^vi^	173.66 (3)	Cs1^vi^—O1—Cs1^xiii^	78.860 (3)
O2^x^—Mg1—Cs1^vi^	81.10 (3)	Cs1^ii^—O1—Cs1^xiii^	174.41 (4)
O1—Mg1—Cs1^ii^	66.55 (3)	Cs1^ix^—O1—Cs1^xiii^	98.45 (4)
O3^viii^—Mg1—Cs1^ii^	64.25 (3)	P1—O2—Mg1^v^	136.32 (7)
O2^ix^—Mg1—Cs1^ii^	81.10 (3)	P1—O2—Cs1	102.59 (4)
O2^x^—Mg1—Cs1^ii^	173.66 (3)	Mg1^v^—O2—Cs1	105.27 (4)
Cs1^vi^—Mg1—Cs1^ii^	92.725 (11)	P1—O2—Cs1^ii^	110.51 (4)
O1—Mg1—Cs1^xi^	173.62 (6)	Mg1^v^—O2—Cs1^ii^	98.78 (3)
O3^viii^—Mg1—Cs1^xi^	67.86 (6)	Cs1—O2—Cs1^ii^	96.94 (3)
O2^ix^—Mg1—Cs1^xi^	74.24 (3)	P1—O3—Mg1^xii^	178.96 (13)
O2^x^—Mg1—Cs1^xi^	74.24 (3)	P1—O3—Cs1^ix^	94.51 (5)
Cs1^vi^—Mg1—Cs1^xi^	109.444 (9)	Mg1^xii^—O3—Cs1^ix^	86.11 (4)
Cs1^ii^—Mg1—Cs1^xi^	109.444 (9)	P1—O3—Cs1^xiii^	94.51 (5)
O1—Mg1—Cs1^xii^	116.54 (5)	Mg1^xii^—O3—Cs1^xiii^	86.11 (4)
O3^viii^—Mg1—Cs1^xii^	137.70 (6)	Cs1^ix^—O3—Cs1^xiii^	106.58 (5)
O2^ix^—Mg1—Cs1^xii^	52.79 (3)	P1—O3—Cs1^xiv^	95.14 (8)
O2^x^—Mg1—Cs1^xii^	52.79 (3)	Mg1^xii^—O3—Cs1^xiv^	83.82 (6)
Cs1^vi^—Mg1—Cs1^xii^	133.015 (6)	Cs1^ix^—O3—Cs1^xiv^	125.67 (3)
Cs1^ii^—Mg1—Cs1^xii^	133.015 (6)	Cs1^xiii^—O3—Cs1^xiv^	125.67 (3)
Cs1^xi^—Mg1—Cs1^xii^	69.840 (9)		

Symmetry codes: (i) *x*, −*y*+1/2, *z*; (ii) −*x*+1, −*y*, −*z*+1; (iii) −*x*+1, *y*+1/2, −*z*+1; (iv) −*x*+1/2, −*y*+1, *z*+1/2; (v) −*x*+1/2, −*y*, *z*+1/2; (vi) −*x*+1, −*y*+1, −*z*+1; (vii) *x*+1/2, *y*, −*z*+3/2; (viii) *x*+1/2, *y*, −*z*+1/2; (ix) −*x*+1/2, −*y*, *z*−1/2; (x) −*x*+1/2, *y*+1/2, *z*−1/2; (xi) *x*, *y*, *z*−1; (xii) *x*−1/2, *y*, −*z*+1/2; (xiii) −*x*+1/2, −*y*+1, *z*−1/2; (xiv) *x*−1/2, *y*, −*z*+3/2.
